# Single-cell transcriptomic profiling and characterization of endothelial progenitor cells: new approach for finding novel markers

**DOI:** 10.1186/s13287-021-02185-0

**Published:** 2021-02-24

**Authors:** Mohamed Essameldin Abdelgawad, Christophe Desterke, Georges Uzan, Sina Naserian

**Affiliations:** 1 Biochemistry & Molecular Biotechnology Division, Chemistry Department, Faculty of Science; Innovative Cellular Microenvironment Optimization Platform (ICMOP), Helwan University, Cairo, Egypt; 2 Inserm UMR-S-MD 1197, Hôpital Paul Brousse - Bâtiment Lavoisier, 12-14 avenue Paul Vaillant Couturier, 94800 Villejuif, France; 3 Paris-Saclay University, Villejuif, France; 4 Inserm UMR-S-MD A9, Hôpital Paul Brousse, Villejuif, France; 5 CellMedEx, Saint Maur des Fossés, France

**Keywords:** Endothelial progenitor cells, Transcriptome analyses, Single-cell RNA-sequencing analyses, Protein-protein interaction network analyses, Multi-parametric flow cytometric analyses

## Abstract

**Background:**

Endothelial progenitor cells (EPCs) are promising candidates for the cellular therapy of peripheral arterial and cardiovascular diseases. However, hitherto there is no specific marker(s) defining precisely EPCs. Herein, we are proposing a new in silico approach for finding novel EPC markers.

**Methods:**

We assembled five groups of chosen EPC-related genes/factors using PubMed literature and Gene Ontology databases. This shortened database of EPC factors was fed into publically published transcriptome matrix to compare their expression between endothelial colony-forming cells (ECFCs), HUVECs, and two adult endothelial cell types (ECs) from the skin and adipose tissue. Further, the database was used for functional enrichment on Mouse Phenotype database and protein-protein interaction network analyses. Moreover, we built a digital matrix of healthy donors’ PBMCs (33 thousand single-cell transcriptomes) and analyzed the expression of these EPC factors.

**Results:**

Transcriptome analyses showed that BMP2, 4, and ephrinB2 were exclusively highly expressed in EPCs; the expression of neuropilin-1 and VEGF-C were significantly higher in EPCs and HUVECs compared with other ECs; Notch 1 was highly expressed in EPCs and skin-ECs; MIR21 was highly expressed in skin-ECs; PECAM-1 was significantly higher in EPCs and adipose ECs. Moreover, functional enrichment of EPC-related genes on Mouse Phenotype and STRING protein database has revealed significant relations between chosen EPC factors and endothelial and vascular functions, development, and morphogenesis, where ephrinB2, BMP2, and BMP4 were highly expressed in EPCs and were connected to abnormal vascular functions. Single-cell RNA-sequencing analyses have revealed that among the EPC-regulated markers in transcriptome analyses, (i) ICAM1 and Endoglin were weekly expressed in the monocyte compartment of the peripheral blood; (ii) CD163 and CD36 were highly expressed in the CD14+ monocyte compartment whereas CSF1R was highly expressed in the CD16+ monocyte compartment, (iii) L-selectin and IL6R were globally expressed in the lymphoid/myeloid compartments, and (iv) interestingly, PLAUR/UPAR and NOTCH2 were highly expressed in both CD14+ and CD16+ monocytic compartments.

**Conclusions:**

The current study has identified novel EPC markers that could be used for better characterization of EPC subpopulation in adult peripheral blood and subsequent usage of EPCs for various cell therapy and regenerative medicine applications.

## Background

Endothelial progenitor cells (EPCs) are heterogeneous population of mononuclear cells (MNCs) that originate and reside in the bone marrow (BM); they are circulating in (mobilized to) the adult peripheral (PB) or umbilical cord blood (UCB) [1]. EPCs have been discovered by Asahara and his coworkers in 1997 [2]. They express endothelial antigens like CD31, von Willebrand factor (vWF), endothelial nitric oxide synthase (eNOS), VE-cadherin, and VEGFR2 [3, 4]. EPCs constitute 1–5% of the total BM cells and > 0.0001–0.01% of PB circulating MNCs [5]. They are implicated in homeostasis, neovascularization, vascular repair, endothelial regeneration, and angiogenesis processes [6]. There are two distinct subpopulations of EPCs: early EPCs which give rise to heterogeneous colonies that appear in culture after 3–5 days; they are obtained by negative selection on fibronectin; they are round cells surrounded by spindle-shaped cells in morphology; they have a slow proliferation and their in vitro growth peak is reached after 2–3 weeks [7–10]. Moreover, early EPCs do not form vascular tubes in vitro but they have a strong paracrine activity (secrete a plethora of angiogenic factors) that contributes effectively to neovascularization [11, 12], they have high expression of both hematopoietic and endothelial markers (VEGFR-2, CD31, vWf, able to uptake acLDL and bind UEA-1) [13, 14], they are most likely derived from hematopoietic stem cells and had a resemblance to myeloid progenitors [15], and hence they are also named “hematopoietic EPCs” [16]. Early EPCs generate the endothelial cell colony-forming units (CFU-ECs) in vitro [8, 17]. Interestingly, early EPCs [18] are also termed circulating angiogenic cells (CACs) [19]. On the other hand, the other subtype of EPCs is termed “late EPCs” [18]; they are more homogenous colonies that appear after 2–4 weeks in culture, they are isolated by positive selection on collagen I, they are elongated cells that form a cobblestone*-*morphology monolayer in vitro which is characteristic of endothelial cells, they could be maintained in culture for ~ 12 weeks (up to 15 passages), and they have higher proliferative and clonogenic potential compared with early EPCs [12, 17, 20]. Moreover, late EPCs could easily form tubular/capillary-like structures in vitro, they possess high vasculogenic and angiogenic potential, and in vivo they could incorporate in the existing endothelium where they form stable vessels and continue to differentiate into mature endothelial cells [17, 21, 22]. Noteworthy, late EPCs are phenotypically similar to mature endothelium, they are present/circulate in both PB and UCB; importantly, they are not only closer to endothelium phenotypically but also by exhibiting no hematopoietic (CD45) or monocyte markers (CD14 and CD115) expression in contrast to early EPCs, whereas they express many endothelial cell (EC) antigens (CD31, VEGFR-2, CD105, CD144, CD146, vWf, CD34, higher eNOS, Tie-2, VE-cadherin, able to uptake acLDL and bind UEA-1) [22, 23]. Collectively, late EPCs are termed “non-hematopoietic EPCs” [16, 24], and thus they are considered the “EPCs” subtype that complies the most with the original endothelial phenotype and functions to be the legitimate endothelial progenitor cells bearing almost all of the endothelial cell characteristics [15]. Further, late EPCs generate in vitro “endothelial colony-forming cells or ECFCs” [25] and they are also called “outgrowth endothelial cells or OECs” [20, 26].

There were a number of proposed combinations of surface antigens for identifying EPCs in human; this include (but not restricted to) CD34+, CD31+, CD133+, VEGFR2+, CD144+, CD146+, CD45−/+, CD14+, VEGFR1+, and FGFR1+ [16, 24, 27].

The vast variation in the surface antigens for EPCs is possibly attributed to identifying different EPCs’ subpopulations at various maturation/differentiation phases. The term “EPCs” has been haphazardly used to refer to both circulating (late EPCs) and cultured cells (ECFCs). In addition, the accumulating literature did not provide one consolidated definition of EPCs nor a specific EPC phenotype or a unified isolation and culture protocol of them. Accordingly, different isolation techniques and culturing methods applied resulted in EPCs with various phenotypes [28]. Therefore, we aimed herein using in silico data to reach a possible novel EPC marker or a combination of markers that could specifically characterize EPCs.

In the current manuscript, we are adding to the already ongoing efforts for the characterization analyses of EPCs by presenting a new approach for finding novel marker(s) of EPCs in peripheral blood.

The up-to-date “-omics,” “gene-expression profiling” or “transcriptomics” is currently the most widely used tool for the characterization and functional analysis of cells; moreover, transcriptomics have provided a better understanding for EPCs’ characterization analyses in an unbiased manner [28].

Large genomic data from large tissue sample collections are difficult to analyze; however, if we use the individual transcriptomic data coming from the tissue-representing or “single-cell” level, this would render mass analysis of bulk single-cell(s) data to be fast and non-tedious [29, 30] and thus would introduce new insights about the ontogeny of new and rare cell types and the relationships between various cell lineages [31]. Collectively, single-cell transcriptomics would help herein to improve our knowledge for the identification and characterization of EPCs in peripheral blood.

Using Gene Ontology and literature survey, we assembled five groups of EPCs’ molecules/factors/markers that have been specifically chosen for being of special interest and importance to the EPC biology.

The categorization and choice of various factors were based on grouping different molecules/factors into groups involved in similar EPC and EC functions. The first group is involved in developmental angiogenesis, tumor angiogenesis, and vascular development; this group comprises neuropilins (NRP1 and NRP2), semaphorins (3A, 3B, 3D, 3E, 3F, 4A, 4D, 5A, and 6A), and VEGFR1, 2, and 3 [32–35]. The second group is implicated in ECs/EPCs-immune cell interaction, proliferation, migration, survival, apoptosis, angiogenesis, immunogenicity, and immune-modulation. It includes TNF-α, TNFR2/P75, TNFR1/P55, and TRAIL (tumor necrosis factor-related apoptosis-inducing ligand) [36–40]. The third group of factors is engaged in proliferation, survival, migration, and differentiation of vascular stem/progenitor cells which includes closely related cells co-inhabiting the vascular niche, namely they are EPCs, smooth muscle cells (SMCs), pericytes, and mesenchymal stem cells (MSCs). The representing candidates of this group were PDGF-(A, B, and C), BMP (2, 4, and 9), Wnt (1, 4, 11, and 5A), VEGF (A and C), TGF β, FGF2, IFG-1, and EGF [41, 42]. Group 4 comprises microRNAs which are small, non-coding, single-stranded RNAs with regulatory activities. Recent studies showed that microRNAs play an important role in regulating EPC functions which include proliferation, senescence, apoptosis and autophagy, mobilization and migration, tube formation and angiogenic capacity, and differentiation. We have chosen representative microRNAs that could be involved in one or more biological processes; the chosen candidates were microRNA-221/222, 34a, 126, 16,107, 150, 22, 21, and 130 a [43–45]. The fifth group is involved in the internalization (of ligands from the extracellular matrix to be recycled back to the endosomal compartment), endocytosis, migratory and/or invasive capacity, and motility. It comprises urokinase plasminogen activator (uPA), urokinase plasminogen activator receptor (uPAR), urokinase plasminogen activator receptor-associated protein (uPARAP), tissue-type plasminogen activator (tPA), Neuropilin-1 NRP1, Neuropilin-2 NRP2, VEGFR1, 2 and 3, PECAM-1, ICAM-1, VE-cadherin, Ephrin-B2, EphB4, and EGFL7 [46–57].

Herein, our main objective is to search for novel markers of EPCs in peripheral blood. Thus, we have created a short list divided into five groups of EPC factors/molecules using PubMed literature, Gene Ontology, and other sources. This list was used for both the transcriptomic and single-cell analyses. In transcriptome analyses, the list was used to compare the relative expression of various EPC genes (involved within this list) between ECFCs, HUVECs, and two adult ECs from the skin and adipose tissue. Moreover, EPC chosen-genes were used for functional enrichment on Mouse Phenotype and STRING protein-protein interaction network database to decipher the involvement of these factors in endothelial and vascular development and morphogenesis. Additionally, we built a digital matrix of healthy donors’ PBMCs (33 thousand transcriptomes) and analyzed the expression of the short list of EPC factors and more specifically EPC molecules that have shown to be significantly regulated between ECFCs and the other three adult ECs in the transcriptome analyses.

The current study has identified novel markers, which include secreted factors, miRNAs, and growth factors. Among these markers we have analyzed, some of them could be used for better cytometric analyses and an optimized characterization of EPC subpopulation in peripheral blood.

## Materials and methods

### Semantic search for chosen factors implicated in recent endothelial progenitor cell biology field

Using Gene Ontology, a vast array of EPCs’ physiology/pathophysiology-related published research and literature and PubMed databases were used in the current work. This was followed by the selection and categorization of different factors (affecting various signaling cascades, molecular functions, and biological processes of EPCs) into five main groups of molecules/factors using a combination of keywords in the field of the EPC biology. The five molecular sets were described in Table 1 with their related employed keywords. We have chosen sixty-one factors distributed as follows: group 1 (purple; 14 molecules), group 2 (green; 4 molecules), group 3 (red; 19 molecules), group 4 (blue; 9 molecules), and group 5 (brown; 15 molecules) as shown in Table 1.

**Table 1:**
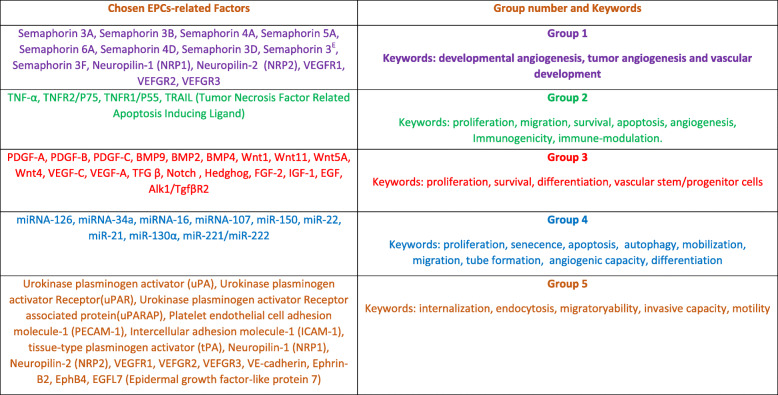
Table comprising semantic determination of molecule sets related to EPC/EC biology. Sixty-one factors distributed as follows: group 1 (purple; 14 molecules), group 2 (green; 4 molecules), group 3 (red; 19 molecules), group 4 (blue; 9 molecules), and group 5 (brown; 15 molecules). The keywords used for each group of molecules are slightly changed between the groups depending on the biological functions that various molecules/factors are incorporated in. It has to be noted that VEGFR1, 2, and 3 were repeated in groups 1 and 5 as they are differently involved in the general molecular functions of each group

### Public datasets

ECFCs and mature ECs have been already studied by whole transcriptome analysis through Gene Omnibus Expression dataset from the series GSE55695 [58]. In these experiments, ECFCs of the peripheral blood (ECFC-PB) were compared to different kinds of endothelial cells: adipose tissue-derived endothelial cells (EC-ADIPO), dermal microvascular endothelial cells (EC-skin), and human umbilical vein endothelial cells (HUVECs). The expression matrix normalized by quantile normalization method was downloaded at the following web address: https://www.ncbi.nlm.nih.gov/geo/query/acc.cgi?acc=GSE55695. In a second step, the normalized matrix was annotated with the corresponding GEO plateform GPL10558 used for microarray technology: Illumina HumanHT-12 V4.0 expression beadchip.

### Transcriptome analyses

Bioinformatics analyses were performed in R software environment version 3.4.1. Unsupervised principal component analysis was performed with FactoMineR R-package [59]. Molecule names from previously described semantic research in topics of endothelial cells/EPCs (see Table 1) were converted in official human gene symbol with HUGO database from HUGO Gene Nomenclature Committee (HGNC consortium) [60]. Expression heatmap was performed with R-package made4 by using unsupervised classification with Euclidean distances [61]. Most variable genes between the transcriptome of the four experimental groups (ECFC-PB, EC-ADIPO, EC-skin, and HUVECs) were defined by performing Fisher one-way analysis of variance (ANOVA) with implementation of 500 permutations in order to perform multi-testing corrections on *p* values with false discovery rate method in genomic suite Mev version 4.9.0 [62]. Functional enrichment on Mouse Phenotype database was performed with ToppGene software suite [63]. Functional enrichment network was performed with Cytoscape standalone software version 3.6.0 [64].

### Single-cell RNA-sequencing analyses

Transcriptome of 33,000 healthy donors’ peripheral blood mononuclear cells (PBMCs) which were found publically available (10X genomics, https://www.10xgenomics.com/solutions/single-cell/) were analyzed to assess the expression of the chosen EPC-related markers in peripheral blood as shown in Table 2. Sequencing reads were analyzed with demultiplexing solution: Cell Ranger version 1.1.0. Seurat algorithm version 2.3.0 [65] was used in R software environment version 3.4.3 to build a digital matrix of the transcriptomes and subsequent clustering by combining principal component analysis and tSNE (t-distribution stochastic neighbor embedding) mathematical reductions in order to project the quantification of the studied endothelial markers.

**Table 2:** Most significant EPC-related genes found by ANOVA between ECFCs and other three types of endothelial cells: most variable EPC-related genes found to be significant by ANOVA between ECFCs (in peripheral blood) and three distinct groups of endothelial cells: HUVECs, adipose, and skin from transcriptome dataset GSE55695. The table shows gene symbol with their relative Illumina identifier, also ratio obtained from the Fisher statistics, and their corresponding corrected *p* value was adjusted for the multi-testing errors

Gene symbol	Description	ID_illumina_DNA_beads	Fisher_F_ratio_ANOVA	Adj. ***p*** value
**VEGFC**	**Vascular endothelial growth factor C**	**ILMN_1701204**	**19.39477**	**0.001**
**BMP4**	**Bone morphogenetic protein 4**	**ILMN_1693749**	**14.54601**	**0.018**
**BMP4**	**Bone morphogenetic protein 4**	**ILMN_1709734**	**10.868284**	**0.004**
**NOTCH2**	**Notch 2**	**ILMN_2405297**	**10.400303**	**0.018**
**BMP4**	**Bone morphogenetic protein 4**	**ILMN_1740900**	**9.894238**	**0.008**
**SEMA3F**	**Semaphorin 3F**	**ILMN_1761540**	**8.470199**	**0.01**
**PLAUR**	**Plasminogen activator, urokinase receptor**	**ILMN_2408543**	**8.119817**	**0.006**
**PDGFA**	**Platelet derived growth factor subunit A**	**ILMN_2342695**	**7.7437563**	**0.018**
**BMP2**	**Bone morphogenetic protein 2**	**ILMN_1722718**	**7.219204**	**0.016**
**PDGFC**	**Platelet-derived growth factor C**	**ILMN_1683023**	**7.012548**	**0.028**
**SEMA6A**	**Semaphorin 6A**	**ILMN_1713529**	**6.958105**	**0.016**
**NOTCH4**	**Notch 4**	**ILMN_1711157**	**6.7292013**	**0.014**
**SEMA3A**	**Semaphorin 3A**	**ILMN_1765641**	**6.5665183**	**0.008**
**PECAM1**	**Platelet and endothelial cell adhesion molecule 1**	**ILMN_1689518**	**6.2686167**	**0.032**
**TNF**	**Tumor necrosis factor**	**ILMN_1728106**	**5.749965**	**0.024**
**NOTCH1**	**Notch 1**	**ILMN_1729161**	**5.5108757**	**0.032**
**PLAUR**	**Plasminogen activator, urokinase receptor**	**ILMN_2374340**	**5.3354907**	**0.008**
**MIR21**	**MicroRNA 21**	**ILMN_3310840**	**5.189098**	**0.036**
**MIR34A**	**MicroRNA 34a**	**ILMN_3308455**	**5.005866**	**0.016**
**NRP1**	**Neuropilin 1**	**ILMN_1742547**	**4.502312**	**0.038**
**EFNB2**	**Ephrin B2**	**ILMN_1703852**	**4.091059**	**0.046**
**SEMA5A**	**Semaphorin 5A**	**ILMN_1880012**	**3.2281258**	**0.026**

### Protein-protein interaction network

Molecular identifiers of EPC selected markers were used to build a protein-protein interaction network with STRING proteomic database [66]. High confident interaction score over 800 was set to select interactions which were validated experimentally. Network Analyst web tool [67] was used to perform functional inference with biological process Gene Ontology database.

### Statistical analysis

Statistical analysis was performed in R software environment version 3.4.1. Statistical hypothesis between groups was verified by performing Fisher one-way analysis of variance with Tukey post hoc test. A significance threshold on alpha error *p* < 0.05 was defined during these analyses.

An overview of the experimental workflow undertaken in the current work is depicted in Fig. 5.

## Results

### Specific transcriptome analyses of endothelial colony-forming cells (ECFCs) compared with other adult endothelial cells revealed a distinct expression profile implicated in abnormal vascular development

In peripheral blood, ECs are derived from endothelial precursors, which are population of cells called endothelial progenitor cells (EPCs). In order to investigate the importance of EPC-affecting molecules/factors in endothelial cells and vascular biology, a semantic research of important chosen molecules/factors was investigated through querying Gene Ontology and PubMed databases with different keywords (Table 1). Merging this database of EPC chosen molecules with annotated transcriptome normalized matrix allowed reducing dimensions of the matrix to 72 Illumina identifiers (data not shown). On this reduced/minimized expression matrix, a Fisher one-way analysis of variance (ANOVA) was performed to compare experimental conditions comprising ECFCs from peripheral blood (ECFC-PB) and three adult types of endothelial cells from different tissues: skin (EC-skin), adipose tissue (EC-ADIPO), and HUVECs. This statistical test performed (with 500 hundred permutations and with corrected *p* value adjusted for the multi-testing errors, threshold adjust *p* value < 0.01) with multi-testing correction identified 19 EPC-related genes which correspond to 22 unique Illumina identifiers (Table 2).

**Fig. 1:**
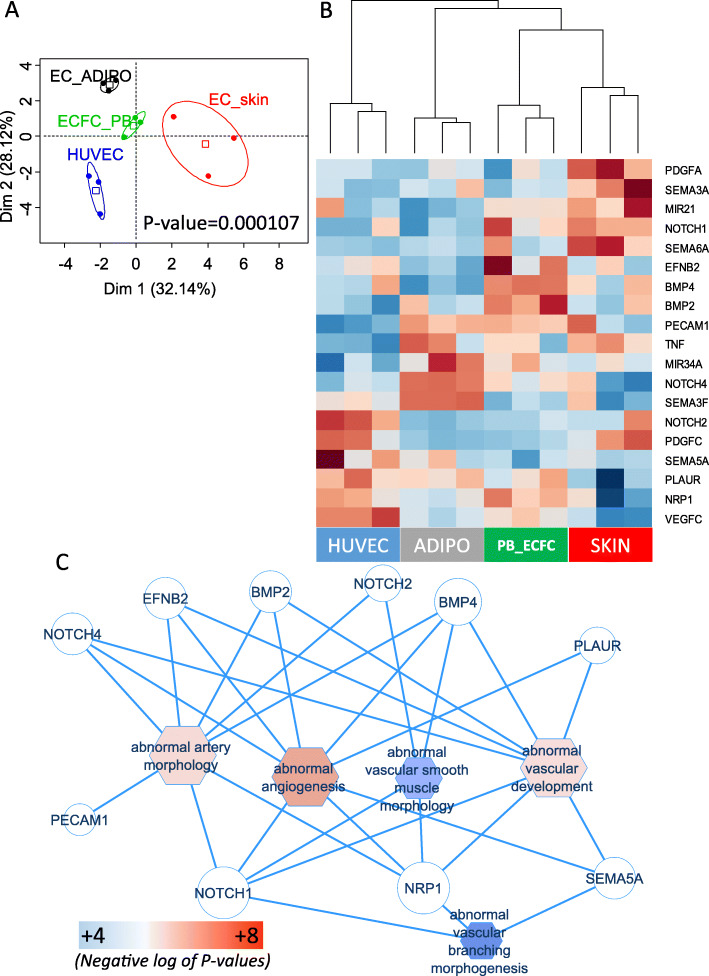
ECFCs compared to other endothelial cells harbored a distinct expression profile implicated in abnormal vascular development. **a** An unsupervised principal component analysis was performed with regulated endothelial-related genes on dataset GSE55695 comparing ECFC_PB (ECFCs in peripheral blood) to distinct groups of endothelial cells (EC_ADIPO, EC_skin and HUVECs, *p* value of group discrimination was calculated on the first principal axis). **b** Expression heatmap of endothelial-related genes performed on transcriptome samples from dataset GSE55695 (unsupervised classification was realized with Euclidean distances with complete method). **c** Functional enrichment network performed with regulated endothelial-related genes in dataset GSE55695 after enrichment on Mouse Phenotype database: circles represent genes; octagons represent enriched function; blue edges represent link(s) between functions and enriched genes; fill color with scale color ranging from blue to red is relative to negative logarithm 10 of the *p* values obtained during the enrichment

Unsupervised principal component analysis performed with the expression of these EPC-related genes significantly discriminate samples through the different experimental conditions (group discrimination based on the principal component map, *p* value = 0.000107, Fig. 1a).

Unsupervised classification (clusters of samples with Euclidean distances and complete method, Fig. 1b) was performed with these significant EPC-related genes confirming the stratification of the samples by their experimental conditions.

Significant high levels of expression of BMP2, BMP4, and EFNB2 were found for ECFC-PB compared with the other three ECs (Fig. 1b). Moreover, significant high levels of expression of MIR34A, NOTCH4, and SEMA3F were found for EC-ADIPO compared with other groups (Fig. 1b). Further, significant high levels of expression of PDGFA and SEMA3A were found for EC-skin compared with other groups (Fig. 1b). The most significant gene found between the four types of cells was VEGF-C (vascular endothelial growth factor C; *p* = 0.001, Table 2) and VEGF-C was found to have a high level of expression specifically in HUVECs (Fig. 1b).

Functional enrichment of EPC-related genes on Mouse Phenotype database allowed finding significant relations between these EPC-related genes and endothelial functions (Table 3). These relations were used to build a functional enrichment network (Fig. 1c): EFNB2, BMP2, and BMP4 molecules were found to have a significant high level of expression exclusively in ECFCs (Fig. 1b) and after functional enrichment were also found to be connected to several enriched endothelial phenotypes, which includes abnormal arterial morphology, abnormal angiogenesis, and also abnormal vascular development (Fig. 1c and Table 3).

**Table 3:** Functional enrichment table performed with EPC-related genes on a database of mouse phenotype. Columns respectively describe the database employed during the functional enrichment, mouse phenotype identifier with their description, and number of genes found to be implicated in the enriched phenotype with respective *p* values for each phenotype (*p* values of enrichment were obtained with Toppgene application)

Database	Mouse phenotype identifiers	Mouse phenotype description	Number of EPC-related genes implicated	***p*** values of enrichment
Mouse Phenotype	MP:0000260	Abnormal angiogenesis	8	2.254E−7
Mouse Phenotype	MP:0002191	Abnormal artery morphology	8	1.953E−6
Mouse Phenotype	MP:0000259	Abnormal vascular development	8	2.402E−6
Mouse Phenotype	MP:0001614	Abnormal blood vessel morphology	10	1.440E−5
Mouse Phenotype	MP:0005602	Decreased angiogenesis	4	2.439E−5
Mouse Phenotype	MP:0005592	Abnormal vascular smooth muscle morphology	4	3.091E−5
Mouse Phenotype	MP:0003227	Abnormal vascular branching morphogenesis	3	5.270E−5

**Fig. 2:**
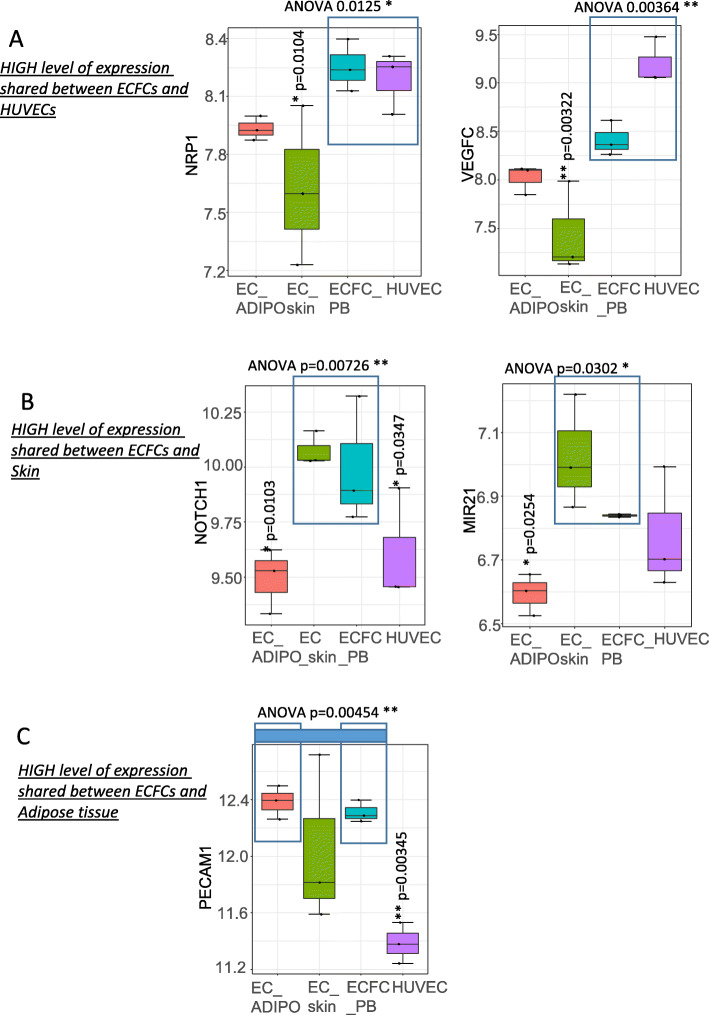
Regulated EPC-related genes sharing elevated level of expression between ECFCs and other three groups of endothelial cells. **a** Genes with a high level of expression shared between ECFCs and HUVECs. **b** Genes with a high level of expression shared between ECFCs and skin endothelial cells. **c** Genes with a high level of expression shared between ECFCs and adipose tissue endothelial cells. The statistical test used to obtain *p* values was performed with one-way ANOVA followed by Tukey post hoc test for multiple comparisons

Some EPC-related genes were also found to have a high level of expression shared between ECFCs and other types of endothelial cells. NRP1 (neuropilin 1) was found to share a high level of expression between ECFCs and HUVECs compared with other groups (ANOVA; *p* value = 0.0125, Fig. 2a) and especially compared with EC-skin (ANOVA; *p* value = 0.0104, Fig. 2a). Moreover, VEGF-C was found to share a high level of expression between ECFCs and HUVECs compared with other groups (ANOVA; *p* value = 0.00364, Fig. 2a) and especially compared with EC-skin. Further, some EPC-related genes also shared a high level of expression between ECFCs and EC-skin (Fig. 2b) which may contribute to cluster ECFCs and EC-skin as near neighbors on the expression heat map (Fig. 1b). NOTCH1 shared a significant high level of expression in ECFCs and EC-skin (*p* value = 0.0073, Fig. 2b), more particularly compared with EC-ADIPO (*p* value = 0.0103, Fig. 2b) and also compared with HUVECs (*p* value = 0.0347, Fig. 2b).

MIR21 was also found to have a significant high level of expression in EC-skin and ECFCs compared with other groups (*p* value = 0.0302, Fig. 2b) and more particularly compared with EC-ADIPO (*p* value = 0.0254, Fig. 2b). One molecule PECAM1, platelet and endothelial cell adhesion molecule 1, was found to share a significant high level of expression between ECFCs and EC-ADIPO (*p* value = 0.00454, Fig. 2c) and more particularly compared with HUVECs (*p* value = 0.00345, Fig. 2c). These results suggest that the EPC chosen molecules that we highlighted during the transcriptomic analyses between different types of endothelial cells are implicated in vascular development and could have an impact on human endothelial phenotype because they are upregulated in these cells.

### Peripheral blood mononuclear cells from healthy donors expressed EPC markers in different sub-compartments characterized by single-cell RNA sequencing

**Fig. 3:**
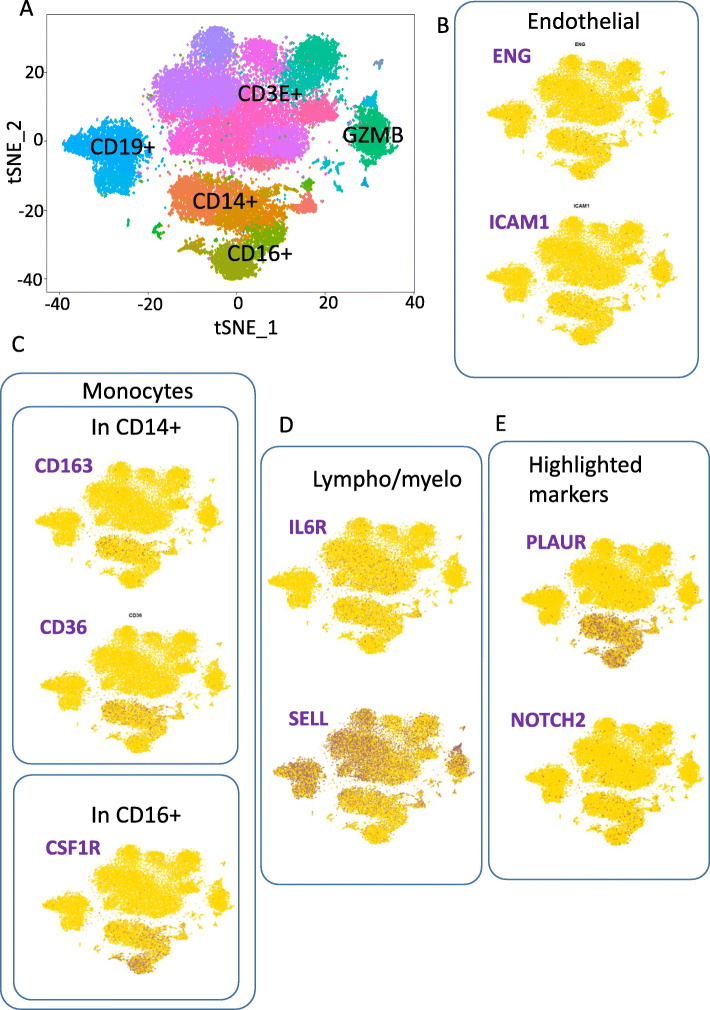
Expression of selected and highlighted EPC-regulated markers in healthy donors’ PBMCs by single-cell RNA sequencing. **a** Cluster identification inside circulating population of 33,000 PBMCs from healthy donor analyzed by single-cell sequencing with Seurat software. **b**–**e** Quantification by single-cell RNA sequencing of molecular markers in healthy donor PBMCs: background of cells with negative expression is colored in gold and positive cells for the markers appeared in dark blue. **b** Expression of endothelial-related markers selected by literature curating. **c** Expression of markers selected by literature curating and were found to have lympho/myeloid expression. **d** Expression of markers selected by literature curating and were found to have an expression in monocytes either in CD16+ subpopulation or in CD14+ subpopulation. **e** Expression of highlighted markers that were found to be regulated previously between endothelial populations of different tissues

One of the actual challenges to improve the isolation protocols and the yield of isolated EPCs from peripheral blood (PB) is upgrading the characterization of EPCs using specific new markers. In this regard, in order to improve the choice of markers for EPC subpopulation, using publicly available single-cell RNA-sequencing experiments, we built a digital matrix of healthy donors’ PBMCs (33,000 single-cell transcriptomes) and analyzed the expression of EPC markers curated from the literature (Table 1) and more particularly EPC markers/genes shown to be highly regulated between EPCs/ECFCs and other ECs from different tissues (Table 2). Seurat algorithm allowed identifying five major cell populations after tSNE mathematical reduction (Fig. 3a): CD19+ cells (B lymphocytes), CD3E+ cells (general T lymphoid marker), Granzyme B cells (natural killer cells and cytotoxic T lymphocytes), CD16+ monocytes, and CD14+ monocytes. In peripheral blood, we assessed the molecular expression of endothelial markers like ICAM1 and ENG, which were at low levels in the monocyte compartment and more particularly in the CD14+ compartment for the ICAM1 expression (Fig. 3b). Other less endothelial-specific markers curated from the literature confirmed the involvement of PB monocyte compartment as the source of ECs/EPCs, principally by the expression of CD163 and CD36 in CD14+ monocyte compartment and also the expression of CSF1R (CD115) in CD16+ monocyte compartment (Fig. 3c). These results suggest the potential implication of EPC subpopulation in monocyte sub-compartment; thus, with the help of the assessed markers, a better understanding of EPC heterogeneities could be achieved. Some EPC genes curated from literature harbored a mixed lympho/myeloid expression in PBMCs; this is the case for SELL (CD62L, selectin L) and IL6R which have a high expression in the lympho/myeloid compartment (Fig. 3d). The latter two markers with elevated expression in the lympho/myeloid compartment, especially CD62L, could be interesting to be used for better EPC characterization, where they could be used as pre-gating endothelial markers on the total population of PBMCs.

Interestingly, among EPC markers that appeared in the transcriptomic analyses (Fig. 1 and Table 2), two of them were found to have a positive expression in PBMCs: PLAUR and NOTCH2 in monocyte compartment (Fig. 3e) either in CD14+ or in CD16+ compartments, with a higher expression of PLAUR. Thus, PLAUR could be also used as EPC marker.

All these results of single-cell RNA-sequencing obtained for EPC-related markers expressed in PBMCs would be useful to design multi-parametric flow cytometric analyses for optimal and better characterization of EPC subpopulation in the peripheral blood.

### EPC markers inferred a molecular network which is implicated in morphogenesis and vascular development

**Fig. 4:**
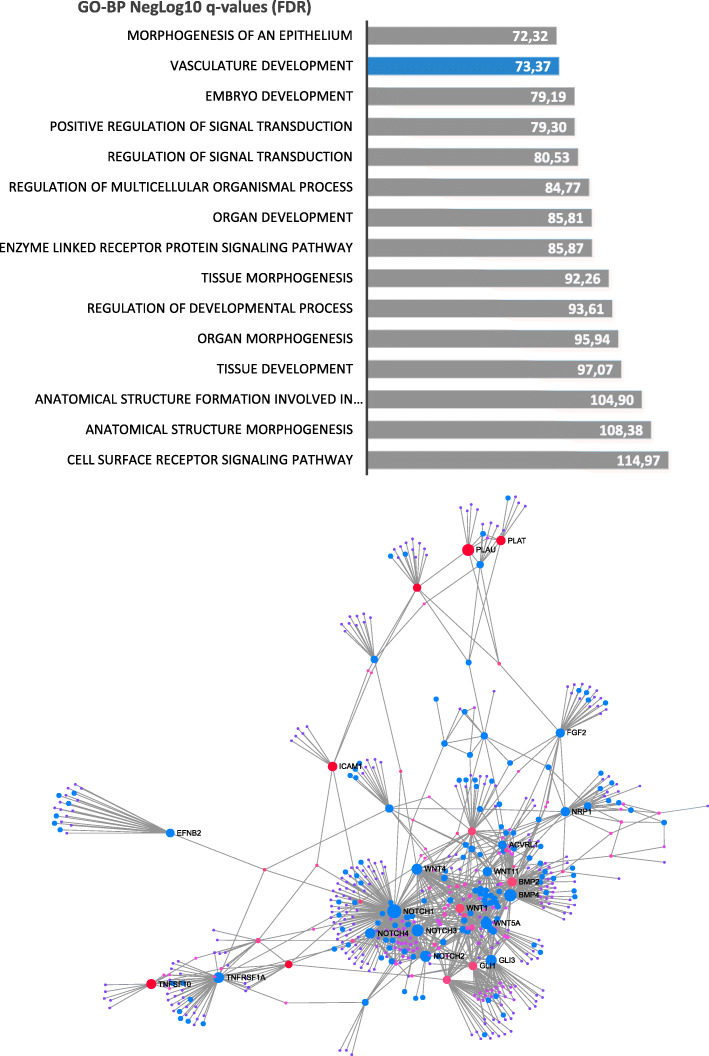
Protein-protein interaction network of EPC selected molecules: protein-protein interaction network built with 42 seeds (red nodes) on string database with stringent parameters (interactions used were experimentally validated); blue nodes represent functional inference of vasculature development found with Gene Ontology biological process

Among the sixty-one EPC markers selected for the study (Table 1), forty-two of them were retained as seeds of the network (red nodes on network, Fig. 4) by STRING protein database with stringent parameters (interaction score over 800 and interaction validated experimentally). Building protein-protein interaction network around these 42 seeds revealed a network comprising a total of 550 nodes with 1086 edges (Fig. 4). Functional inference on this interaction network with Biological Process (Gene Ontology) database revealed an important involvement of these molecule partners in morphogenesis (figure network, barplot) and also their implication in vascular development (blue nodes on network and blue bar in the barplot, Fig. 4 network). These results confirmed that the EPC-related markers that we have selected for this study could influence morphogenesis and vascular development processes.

## Discussion

**Fig. 5:**
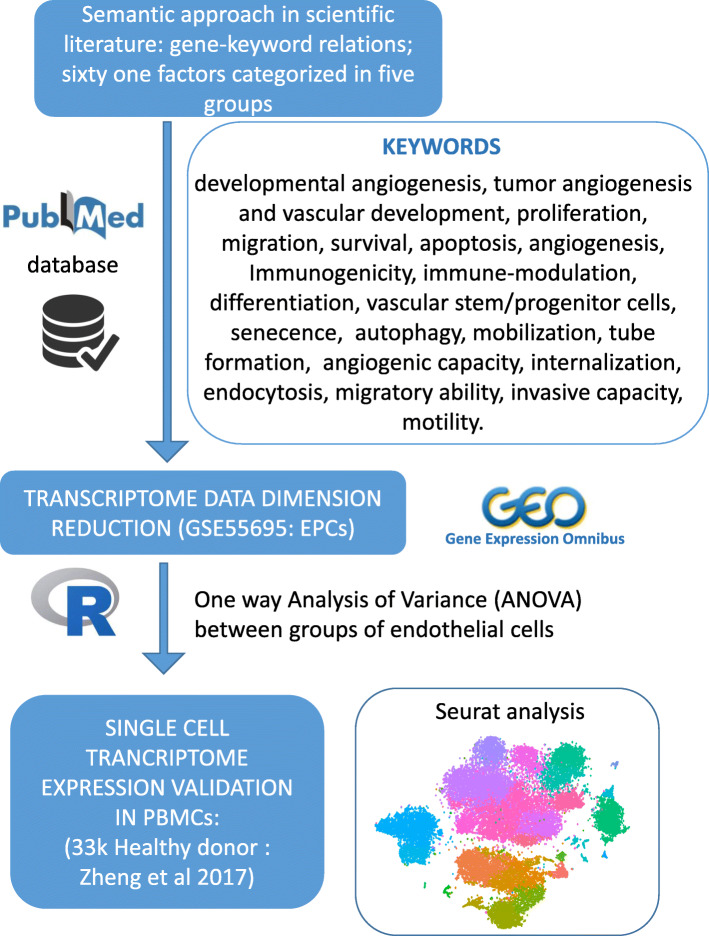
Experimental workflow of the work. The figure shows the hierarchy of the experimental work in the current project

Since the discovery of endothelial progenitor cells (EPCs) three decades ago, there is/are no definitive/globally agreed upon marker or group of markers for the specific molecular characterization of EPCs. Thus, in the current work, we propose a novel in silico approach for finding novel markers of EPCs. We investigated the importance of sixty-one EPC-affecting molecules/factors in EPCs and vascular biology; we conducted semantic research of the chosen molecules/factors curated from the literature via querying Gene Ontology and PubMed databases with different keywords (Fig. 5). Merging these databases of EPC markers into publically available annotated transcriptome normalized matrix to compare the expression of these chosen EPC genes between ECFCs, HUVECs, and two adult ECs from the skin and adipose tissue has revealed that BMB2, BMP4, and EFNB2 (Ephrin B2) have significantly higher expression compared with other groups. Erythropoietin-producing human hepatocellular carcinoma (ephrin) receptors like Ephrin B2 are expressed by ECs [68] and EPCs [69], and they are important for embryonic angiogenesis, cellular adhesion, and migration [70]. Moreover, preconditioning EPCs with Ephrin B2 increases their angiogenic capacity in the hind limb model [71] and in wound healing [72].

Our transcriptomic analysis has showed that both BMB2 and BMP4 are also upregulated in ECFCs. It has been demonstrated that both BMP2 and BMP4 were exclusively expressed by late EPCs (ECFCs) and they are essential for the angiogenic potential of ECFCs [73]. Moreover, BMP4 is implicated in endothelial lineage differentiation of embryonic pluripotent cells [74, 75].

Further, BMP2 could enhance the vasculogenic differentiation of ECFCs co-encapsulated with mesenchymal stromal cells in synthetic scaffold [76]. Interestingly, the same three EPC molecules were the highest significantly regulated genes in the mouse functional enrichment network. Collectively, this means that EFNB2, BMB2, and BMP4 are crucial for ECFC commitment to the endothelial lineage and they are involved in the angiogenic capacity of ECFCs.

Some molecules have shown a high level of expression between ECFCs and HUVECs; NRP1 shared a high level of expression between ECFCs and HUVECs compared with other groups. NRP1 was proved to orchestrate the committed differentiation of endothelial precursors for both human and murine embryonic stem cells [77]. Moreover, it regulates the differentiation of murine pluripotent stem cells to vascular progenitor cells [78], and it is in generally important for angiogenesis and homeostasis [79].

VEGF-C was also upregulated in both ECFCs and HUVECs; it is the most regulated gene with a high level of expression in both HUVECs and ECFCs and it is known to promote lymphatic endothelial cells from human pluripotent stem cells [80]. Moreover, VEGF-C induced the differentiation of lymphatic endothelial progenitor cells (LEPCs) into lymphatic ECs, and it also boosted their incorporation in the cardiac lymphatic system and thus VEGF-C stimulated cardiac lymphangiogenesis in a rat model of myocardial infarction [81].

Whereas the expression of other molecules was elevated in both ECFCs and skin endothelial cells, this includes NOTCH1 and MIR21. NOTCH1 via downstream action on HES1 influenced switch of hematopoietic versus endothelial fate specification [82]. Further, NOTCH1 regulates the differentiation of mouse embryonic stem cells into arterial ECs and increases the angiogenic potential of them [83]. MIR21 induces EPC proliferation [84], and it also modulates their senescence [85]. Additionally, MIR21 is known to have a protective effect on vascular ECs [86].

On the other hand, PECAM1 has shown a shared high level of expression between ECFCs and adipose tissue endothelial cells. PECAM1 is a classical marker of adult ECs so it is not surprising to be upregulated in adipose-derived ECs and it has also been reported to be a maker of ECFCs [17, 27]. Thus, it can be concluded that there was a high level of expression of the chosen factors in ECFCs as compared to other endothelial cells.

The functional enrichment of our chosen sixty-one EPC-related factors on Mouse Phenotype database has shown the significant involvement of the chosen EPC factors, specifically EFNB2, BMB2, and BMP4 which have the highest significant upregulation in ECFCs compared with other groups in the transcriptomic analyses, in mouse endothelial phenotypes like abnormal blood vessel morphology (with the highest number of EPC-related genes involved), followed by abnormal vascular development, abnormal artery morphology, and also decreased angiogenesis (Table 3). Interestingly, the mouse functional enrichment analyses were consistent with the STRING analysis of functional protein-protein interaction networks, which revealed the involvement of 42 out of the chosen molecules as seeds of the network and they were crucial for vascular morphogenesis and vascular development (Fig. 4). Collectively, these results clearly prove the prominence of our chosen EPC-related factors and that they are crucial for endothelial and vascular physiology and pathophysiology.

There are two major types of blood for isolation of EPCs, namely the umbilical cord blood (UCB) and peripheral blood (PB). Although PB is the most available source, however, the number of EPCs and the probability of having EPC colonies from PB is much lower compared with UCB [5, 87]. Thus, herein, our single transcriptomic analyses derived from 33,000 single-cell transcriptomes of healthy donor PBMCs have revealed that EC markers like ICAM1/CD54 (activated EPCs marker) and ENG (Endolgin/CD105) were still expressed at low levels at the monocytic compartments of PB, although the previous markers are authentically established markers of both ECs and EPCs [17, 27].

Further, other EPC markers like CD163, CD36, and CD115 have been shown to be expressed in the monocytic compartment of PB, namely CD163 and CD36 EPCs in the CD14+ monocyte compartment and CSF1R (CD115) in the CD16+ monocyte compartment (Fig. 3c). Noteworthy is that both CD163 [27] and CD115 [17] are considered markers for early EPCs, whereas CD36 [27] is attributed as a late EPC marker. Hence, this proves the existence/the involvement of EPCs as a subpopulation of the monocytic PB sub-compartment. Collectively, the latter EPC markers could improve the study of EPC ontogeny and heterogeneities in PB and will also aid (when used with other conventional markers of EPCs) in better characterization, isolation, and higher yield of EPC colonies from PB.

Other less curated EPC markers from the literature have demonstrated high mixed lympho/myeloid expression in PBMCs which is the case of SELL (CD62L, selectin L); it has been demonstrated that CD62L has been expressed by EPCs, and it is even used as a marker for isolation and characterization of EPCs in combination with CD34 [27].

The same holds true for IL6R which has less expression in lympho/myeloid compartments of PBMC compared with CD62L. Actually, IL6R/CD126/gb80 is an indirect marker of activated ECs/EPCs, as IL6R is not expressed by ECs but it is expressed by neutrophils and monocytes. Moreover, IL6R is proteolytically cleaved forming a complex with IL6, and such complex binds with the gp130 receptor which is expressed ubiquitously on ECs to be activated and then they start expressing ICAM1, VCAM1, and IL6 [88]. We could conclude that the previous two markers with high expression in the lympho/myeloid compartment, especially CD62L, could be used as EPC markers for better characterization and isolation of EPCs from PBMC population.

Interestingly, the same two EPC-related gene markers, namely PLAUR and NOTCH2 that have been shown to be highly regulated between EPCs and other ECs from different tissues (Fig. 1 and Table 2), have also been shown in our single-cell RNA-sequencing analyses to be highly expressed in PBMC monocyte sup-compartment (Fig. 3e) either in CD14+ or in CD16+ sup-compartments, where PLAUR has a much higher expression. UPAR/PLAUR/CD87 is the receptor of UPA and both of them in addition to uPARAP form the UPA/UPAR/uPARAP system. This system is involved in the migration, proliferation, and adhesion of cells. Moreover, this system is a key orchestrator of angiogenesis besides other cellular processes that include receptor shedding and internalization, protein expression, phenotype modulation and tissue remodeling, cancer progression, and metastasis [47, 51, 53–55]. In order for angiogenesis to occur, EPCs have to be released from the basement membrane then they migrate to distant regions where there is injury or neovascularization. UPA binds to UPAR on EC/EPC surface resulting in the formation of plasmin (activation or conversion of plasminogen to plasmin) which activates matrix metalloproteinases (MMPs) like MMP-3 and MMP-12 that in turn cleaves basement membrane releasing EPCs free to migrate and recruited to sites where neovascularization occurs where they differentiate progressively to mature ECs; moreover, MMPs also release growth factors like VEGF, FGF2, and HGF which activate the proliferation of EPCs [89]. Additionally, it has been shown that EPCs showed higher uPAR levels and uPA activity compared with mature ECs [90]. Adding to this, UPAR is a crucial pro-angiogenic regulator for ECFCs and it is also inducing VEGF activity [91]. Also, it has been shown that UPAR-CD36 interaction is important for the pathogenesis of atherosclerosis [92]. Collectively, UPAR/PLAUR has been proven to be a key player in angiogenesis, vasculogenesis, and EPC function and physiology. To summarize, in the current study, we are introducing a novel set of EPC markers (which include secreted factors, miRNAs, and growth factors), where we would propose a novel combination of conventional EC/EPC markers (like CD31, VEGFR2 (KDR), and vWF) and novel EPC markers emerging from the current study, like UPAR/PLAUR and CD36, as plausible panel of markers to be used for EPCs pre-gating on total PBMC population to design multi-parametric flow cytometric analyses and thus would aid in an improved characterization, isolation, and higher yield of EPC colonies from peripheral blood.

## Conclusions

In conclusion, we report a new single-cell transcriptomic in silico approach for delineating a novel characterization panel of novel EPC markers that would help to design a multi-parametric cytometric analyses for optimal and better characterization of EPC subpopulation in peripheral blood and thus improving the isolation and yield of EPCs from peripheral blood for the subsequent use of EPCs in cell therapy and regenerative medicine applications.

## Data Availability

All data generated or analyzed during this study are included in this published article.

## Abbreviations

EPCs: Endothelial progenitor cells
MNCs: Mononuclear cells
BM: Bone marrow
PB: Peripheral blood
UCB: Umbilical cord blood
vWF: von Willebrand factor
eNOS: Endothelial nitric oxide synthase
UEA-1: *Ulex europaeus* agglutinin I
ECFCs: Endothelial colony-forming cells
uPA: Urokinase plasminogen activator
uPAR: Urokinase plasminogen activator receptor
uPARAP: Urokinase plasminogen activator receptor-associated protein
PECAM-1: Platelet endothelial cell adhesion molecule-1
ICAM-1: Intercellular adhesion molecule-1
tPA: Tissue-type plasminogen activator
NRP1: Neuropilin-1
NRP2: Neuropilin-2
VEGFR1: Vascular endothelial growth factor receptor 1
VEFGR2: Vascular endothelial growth factor receptor 2
VEFGR3: Vascular endothelial growth factor receptor 3
EGFL7: Epidermal growth factor-like protein 7
TNFR1/P55: Tumor necrosis factor receptor type 1 (p55)
TNFR2/P75: Tumor necrosis factor receptor type 2 (p75)
TRAIL: Tumor necrosis factor-related apoptosis-inducing ligand
LEPCs: Lymphatic endothelial progenitor cells
PBMCs: Peripheral blood mononuclear cells
tSNE: t-distribution stochastic neighbor embedding
HES1: Hairy and enhancer of split 1
MPPs: Matrix metalloproteinases
HUVECs: Human umbilical vein endothelial cells

## References

[1] Ribatti D, Nico B, Crivellato E, Vacca A (2005). Endothelial progenitor cells in health and disease. Histol Histopathol.

[2] Asahara T, Murohara T, Sullivan A, Silver M, van der Zee R, Li T (1997). Isolation of putative progenitor endothelial cells for angiogenesis. Science..

[3] Loomans CJ, Wan H, de Crom R, van Haperen R, de Boer HC, Leenen PJ (2006). Angiogenic murine endothelial progenitor cells are derived from a myeloid bone marrow fraction and can be identified by endothelial NO synthase expression. Arterioscler Thromb Vasc Biol.

[4] Hristov M, Erl W, Weber PC (2003). Endothelial progenitor cells: mobilization, differentiation, and homing. Arterioscler Thromb Vasc Biol.

[5] Khan SS, Solomon MA, McCoy JP (2005). Detection of circulating endothelial cells and endothelial progenitor cells by flow cytometry. Cytometry B Clin Cytom.

[6] Zhang M, Rehman J, Malik AB (2014). Endothelial progenitor cells and vascular repair. Curr Opin Hematol.

[7] Liew A, Barry F, O’Brien T (2006). Endothelial progenitor cells: diagnostic and therapeutic considerations. Bioessays.

[8] Shantsila E, Watson T, Tse H-F, Gregory YH (2008). New insights on endothelial progenitor cell subpopulations and their angiogenic properties. J Am Coll Cardiol.

[9] Williamson K, Stringer SE, Alexander EY (2012). Endothelial progenitor cells enter the aging arena. Front Physiol.

[10] Hill JM, Zalos G, Halcox JPJ, Schenke WH, Waclawiw MA, Quyyumi AA (2003). Circulating endothelial progenitor cells, vascular function, and cardiovascular risk. N Engl J Med.

[11] Mukai N, Akahori T, Komaki M, Li Q, Kanayasu-Toyoda T, Ishii-Watabe A (2008). A comparison of the tube forming potentials of early and late endothelial progenitor cells. Exp Cell Res.

[12] Sieveking DP, Buckle A, Celermajer DS, MKC Ng. Strikingly different angiogenic properties of endothelial progenitor cell subpopulations: insights from a novel human angiogenesis assay. J Am Coll Cardiol 2008;51(6):660–8. 10.1016/j.jacc.2007.09.059. 10.1016/j.jacc.2007.09.059 18261686

[13] Kaushal S, Amiel GE, Guleserian KJ, Shapira OM, Perry T, Sutherland FW (2001). Functional small diameter neovessels created using endothelial progenitor cells expanded ex vivo. Nat Med.

[14] Reyes M, Dudek A, Jahagirdar B, Koodie L, Marker PH, Verfaillie CM (2002). Origin of endothelial progenitors in human postnatal bone marrow. J Clin Invest.

[15] Grochot-Przeczek A, Kozakowska M, Dulak J, Jozkowicz A, Dulak J, Jozkowicz A, Loboda A (2013). Endothelial cell origin, differentiation, heterogeneity and function. Angiogenesis and vascularisation cellular and molecular mechanisms.

[16] Alexandru N, Titorencu I, Frunza S, Weiss E, Badila E, Georgescu A, Kartha CC, Ramachandran S, Pillai RM (2017). Endothelial progenitor cell dysfunction in the pathogenesis of vascular complications of diabetes. Mechanisms of vascular defects in diabetes mellitus.

[17] Yoder MC, Mead LE, Prater D, Krier TR, Mroueh KN, Li F (2007). Redefining endothelial progenitor cells via clonal analysis and hematopoietic stem/progenitor cell principals. Blood..

[18] Hur J, Yoon CH, Kim HS, Choi JH, Kang HJ, Hwang KK (2004). Characterization of two types of endothelial progenitor cells and their different contributions to neovasculogenesis. Arterioscler Thromb Vasc Biol.

[19] Rehman J, Li J, Orschell CM, March KL (2003). Peripheral blood “endothelial progenitor cells” are derived from monocyte/macrophages and secrete angiogenic growth factors. Circulation.

[20] Lin Y, Weisdorf DJ, Solovey A, Hebbel RP (2000). Origins of circulating endothelial cells and endothelial outgrowth from blood. J Clin Invest.

[21] Asahara T, Kawamoto A, Masuda H (2011). Concise review: circulating endothelial progenitor cells for vascular medicine. Stem Cells.

[22] Prater DN, Case J, Ingram DA, Yoder MC (2007). Working hypothesis to redefine endothelial progenitor cells. Leukemia..

[23] Bouvard C, Gafsou B, Dizier B, Galy-Fauroux I, LokajczykA B-VC (2010). alpha6-integrin subunit plays a major role in the proangiogenic properties of endothelial progenitor cells. Arterioscler Thromb Vasc Biol.

[24] Chopra H, Hung MK, Kwong DL, Zhang CF, Pow EHN (2018). Insights into endothelial progenitor cells: origin, classification, potentials, and prospects. Stem Cells Int.

[25] Ingram DA, Mead LE, Tanaka H, Meade V, Fenoglio A, Mortell K, Pollok K (2004). Identification of a novel hierarchy of endothelial progenitor cells using human peripheral and umbilical cord blood. Blood..

[26] Gulati R, Jevremovic D, Peterson TE, Chatterjee S, Shah V, Vile RG (2003). Diverse origin and function of cells with endothelial phenotype obtained from adult human blood. Circ Res.

[27] Can A, Dastouri MR. Endothelial progenitor cells (EPCs) and their function in physiological states.In: Engin AB, Engin A, editors. Endothelium molecular aspects of metabolic disorders. CRC Press; 2013. p. 136–150

[28] Gremmels H, Fledderus JO, van Balkom BWM, Verhaar MC (2011). Transcriptome analysis in endothelial progenitor cell biology. Antioxid Redox Signal.

[29] Tang F, Barbacioru C, Wang Y, Nordman E, Lee C, Xu N (2009). mRNA-Seq whole-transcriptome analysis of a single cell. Nat Methods.

[30] Wills QF, Livak KJ, Tipping AJ, Enver T, Goldson AJ, Sexton DW, Holmes C. Single-cell gene expression analysis reveals genetic associations masked in whole-tissue experiments 2013;31(8):748–52. 10.1038/nbt.2642. 10.1038/nbt.2642 23873083

[31] Guo M, Xu Y (2018). Single-cell transcriptome analysis using SINCERA pipeline. Methods Mol Biol.

[32] Klagsbrun M, Takashima S, Mamluk R. The role of neuropilin in vascular and tumor biology. In: Bagnard D, editors. Neuropilin: from nervous system to vascular and tumor biology. Springer Publishers; 2002. p. 33–48. 10.1007/978-1-4615-0119-0_3 12613541

[33] Kumanogoh A, Kikutani H (2013). Immunological functions of the neuropilins and plexins as receptors for semaphorins. Nat Rev Immunol.

[34] Gu C, Giraudo E (2013). The role of semaphorins and their receptors in vascular development and cancer. Exp Cell Res.

[35] Brash JT, Lampropoulou A, Ruhrberg C, Neufeld G, Kessler O (2017). The role of the neuropilins in developmental angiogenesis. The neuropilins: role and function in health and disease.

[36] Kwon YW, Heo SC, Jeong GO, Yoon JW, Mo WM, Lee MJ (2013). Tumor necrosis factor-α-activated mesenchymal stem cells promote endothelial progenitor cell homing and angiogenesis. Biochim Biophys Acta.

[37] Prisco AR, Hoffmann BR, Kaczorowski CC, McDermott-Roe C, Stodola TJ, Exner EC (2016). Tumor necrosis factor α regulates endothelial progenitor cell migration via CADM1 and NF-kB. Stem Cells.

[38] Sasi SP, Song J, Park D, Enderling H, McDonald JT, Gee H (2014). TNF-TNFR2/p75 signaling inhibits early and increases delayed nontargeted effects in bone marrow-derived endothelial progenitor cells. J Biol Chem.

[39] D’Auria F, Centurione L, Centurione MA, Angelini A, Di Pietro R (2015). Tumor necrosis factor related apoptosis inducing ligand (Trail) in endothelial response to biomechanical and biochemical stresses in arteries. J Cell Biochem.

[40] Naserian S, Abdelgawad ME, Bakshloo MA, Ha G, Arouche N, Cohen JL (2020). The TNF/TNFR2 signaling pathway is a key regulatory factor in endothelial progenitor cell immunosuppressive effect. Cell Commun Signal.

[41] Lu W, Li X (2018). Vascular stem/progenitor cells: functions and signaling pathways. Cell Mol Life Sci.

[42] Lu J, Pompili VJ, Das H, Mehta JL, Mathur P, Dhalla NS (2017). Vascular stem cells in regulation of angiogenesis. Biochemical basis and therapeutic implications of angiogenesis, advances in biochemistry in health and disease.

[43] Qu K, Wang Z, Lin X-L, Zhang K, He X-L, Zhang H (2015). MicroRNAs: key regulators of endothelial progenitor cell functions. Clin Chim Acta.

[44] Yamakuchi M, Hashiguchi T. Endothelial cell aging: how miRNAs contribute? J Clin Med. 2018;7(170). 10.3390/jcm7070170. 10.3390/jcm7070170 PMC6068727 29996516

[45] Li X, Chang Y, Ding Z, Guo Z, Mehta JL, Wang X, Mehta JL, Mathur P, Dhalla NS (2017). Functions of microRNAs in angiogenesis. Biochemical basis and therapeutic implications of angiogenesis, advances in biochemistry in health and disease.

[46] Bochenek ML, Dickinson S, Astin JW, Adams RH, Nobes CD (2010). Ephrin-B2 regulates endothelial cell morphology and motility independently of Eph-receptor binding. J Cell Sci.

[47] Sheikh H, Yarwood H, Ashworth A, Isacke CM (2000). Endo180, an endocytic recycling glycoprotein related to the macrophage mannose receptor is expressed on fibroblasts, endothelial cells and macrophages and functions as a lectin receptor. J Cell Sci.

[48] Schmidt M, Paes K, De Mazière A, Smyczek T, Yang S, Gray A (2007). EGFL7 regulates the collective migration of endothelial cells by restricting their spatial distribution. Development..

[49] Cao G, O'Brien CD, Zhou Z, Sanders SM, Greenbaum JN, Makrigiannakis A (2002). Involvement of human PECAM-1 in angiogenesis and in vitro endothelial cell migration. Am J Physiol Cell Physiol.

[50] Kevil CG, Orr AW, Langston W, Mickett K, Murphy-Ullrich J, Patel RP (2004). Intercellular adhesion molecule-1 (ICAM-1) regulates endothelial cell motility through a nitric oxide-dependent pathway. J Biol Chem.

[51] Montuori N, Ragno P (2014). Role of uPA/uPAR in the modulation of angiogenesis. Chem Immunol Allergy.

[52] Narazaki M, Segarra M, Hou X, Tanaka T, Li X, Tosato G (2010). Oligo-guanosine nucleotide induces neuropilin-1 internalization in endothelial cells and inhibits angiogenesis. Blood..

[53] Mondino A, Blasi F (2004). uPA and uPAR in fibrinolysis, immunity and pathology. Trends Immunol.

[54] Del Rosso M (2011). uPAR in angiogenesis regulation. Blood..

[55] Durré T, Morfoisse F, Erpicum C, Ebroin M, Blacher S, García-Caballero M (2018). uPARAP/Endo180 receptor is a gatekeeper of VEGFR-2/VEGFR-3 heterodimerisation during pathological lymphangiogenesis. Nat Commun.

[56] Cao J, Ehling M, März S, Seebach J, Tarbashevich K, Sixta T, et al. Polarized actin and VE-cadherin dynamics regulate junctional remodelling and cell migration during sprouting angiogenesis. Nat Commun. 2017;8(2210). 10.1038/s41467-017-02373-8 10.1038/s41467-017-02373-8 PMC5738342 29263363

[57] Breuss JM, Uhrin P (2012). VEGF-initiated angiogenesis and the uPA/uPAR system. Cell Adhes Migr.

[58] Szöke K, Reinisch A, Østrup E, P Reinholt F, Brinchmann JE. Autologous cell sources in therapeutic vasculogenesis: in vitro and in vivo comparison of endothelial colony-forming cells from peripheral blood and endothelial cells isolated from adipose tissue. Cytotherapy. 2016;18(2):242–52. 10.1016/j.jcyt.2015.10.009. 10.1016/j.jcyt.2015.10.009 26669908

[59] Lê S, Josse J, Husson F (2008). FactoMineR: an R package for multivariate analysis. J Stat Softw.

[60] Yates B, Braschi B, Gray KA, Seal RL, Tweedie S, Bruford EA (2017). Genenames.org: the HGNC and VGNC resources in 2017. Nucleic Acids Res.

[61] Culhane AC, Thioulouse J, Perrière G, Higgins DG (2005). MADE4: an R package for multivariate analysis of gene expression data. Bioinforma Oxf Engl.

[62] Saeed AI, Sharov V, White J, Li J, Liang W, Bhagabati N (2003). TM4: a free, open-source system for microarray data management and analysis. BioTechniques..

[63] Chen J, Bardes EE, Aronow BJ, Jegga AG (2009). ToppGene Suite for gene list enrichment analysis and candidate gene prioritization. Nucleic Acids Res.

[64] Cline MS, Smoot M, Cerami E, Kuchinsky A, Landys N, Workman C (2007). Integration of biological networks and gene expression data using Cytoscape. Nat Protoc.

[65] Butler A, Hoffman P, Smibert P, Papalexi E, Satija R (2018). Integrating single-cell transcriptomic data across different conditions, technologies, and species. Nat Biotechnol.

[66] Szklarczyk D, Franceschini A, Wyder S, Forslund K, Heller D, Huerta-Cepas J (2015). STRING v10: protein-protein interaction networks, integrated over the tree of life. Nucleic Acids Res.

[67] Xia J, Gill EE, Hancock REW (2015). NetworkAnalyst for statistical, visual and network-based meta-analysis of gene expression data. Nat Protoc.

[68] Dodelet VC, Pasquale EB (2000). Eph receptors and ephrin ligands: embryogenesis to tumorigenesis. Oncogene..

[69] Tan W, Wang J, Zhou F, Gao L, Yin R, Liu H (2017). Coexistence of Eph receptor B1 and ephrin B2 in port-wine stain endothelial progenitor cells contributes to clinicopathological vasculature dilatation. Br J Dermatol.

[70] Salajegheh A, Salajegheh A (2016). Erythropoietin-producing hepatocellular receptors B: Ephrin B2, Ephrin B4. Angiogenesis in health, disease and malignancy Springer.

[71] Foubert P, Silvestre J-S, Souttou B, Barateau V, Martin C, Ebrahimian TG (2007). PSGL-1-mediated activation of EphB4 increases the proangiogenic potential of endothelial progenitor cells. J Clin Invest.

[72] Foubert P, Squiban C, Holler V, Buard V, Dean C, Levy BI (2015). Strategies to enhance the efficiency of endothelial progenitor cell therapy by Ephrin B2 pretreatment and coadministration with smooth muscle progenitor cells on vascular function during the wound-healing process in irradiated or nonirradiated condition. Cell Transplant.

[73] Smadja DM, Bièche I, Silvestre J-S, Germain S, Cornet A, Laurendeau I (2008). Bone morphogenetic proteins 2 and 4 are selectively expressed by late outgrowth endothelial progenitor cells and promote neoangiogenesis. Arterioscler Thromb Vasc Biol.

[74] Bai H, Gao Y, Arzigian M, Wojchowski DM, Wu W-S, Wang ZZ (2010). BMP4 regulates vascular progenitor development in human embryonic stem cells through a Smad-dependent pathway. J Cell Biochem.

[75] Goldman O, Feraud O, Ponio JB-D, Driancourt C, Clay D, Le Bousse-Kerdiles M-C (2009). A boost of BMP4 accelerates the commitment of human embryonic stem cells to the endothelial lineage. Stem Cells.

[76] Barati D, Shariati SRP, Moeinzadeh S, Melero-Martin JM, Khademhosseini A, Jabbari E (2016). Spatiotemporal release of BMP-2 and VEGF enhances osteogenic and vasculogenic differentiation of human mesenchymal stem cells and endothelial colony-forming cells co-encapsulated in a patterned hydrogel. J Control Release.

[77] Cimato T, Beers J, Ding S, Ma M, McCoy JP, Boehm M (2009). Neuropilin-1 identifies endothelial precursors in human and murine embryonic stem cells before CD34 expression. Circulation.

[78] Kim D, Lee V, Dorsey TB, Niklason LE, Gui L, Dai G (2018). Neuropilin-1 mediated arterial differentiation of murine pluripotent stem cells. Stem Cells Dev.

[79] Plein A, Fantin A, Ruhrberg C (2014). Neuropilin regulation of angiogenesis, arteriogenesis, and vascular permeability. Microcirculation.

[80] Lee S-J, Park C, Lee JY, Kim S, Kwon PJ, Kim W (2015). Generation of pure lymphatic endothelial cells from human pluripotent stem cells and their therapeutic effects on wound repair. Sci Rep.

[81] Zhang H-F, Wang Y-L, Tan Y-Z, Wang H-J, Tao P, Zhou P (2019). Enhancement of cardiac lymphangiogenesis by transplantation of CD34 + VEGFR-3 + endothelial progenitor cells and sustained release of VEGF-C. Basic Res Cardiol.

[82] Lee JB, Werbowetski-Ogilvie TE, Lee J-H, McIntyre BAS, Schnerch A, Hong S-H (2013). Notch-HES1 signaling axis controls hemato-endothelial fate decisions of human embryonic and induced pluripotent stem cells. Blood..

[83] Park JK, Lee TW, Do EK, Moon HJ, Kim JH (2018). Role of Notch1 in the arterial specification and angiogenic potential of mouse embryonic stem cell-derived endothelial cells. Stem Cell Res Ther.

[84] Du X, Hong L, Sun L, Sang H, Qian A, Li W (2019). miR-21 induces endothelial progenitor cells proliferation and angiogenesis via targeting FASLG and is a potential prognostic marker in deep venous thrombosis. J Transl Med.

[85] Zhu S, Deng S, Ma Q, Zhang T, Jia C, Zhuo D (2013). MicroRNA-10A* and MicroRNA-21 modulate endothelial progenitor cell senescence via suppressing high-mobility group A2. Circ Res.

[86] Xu X, Jiao X, Song N, Luo W, Liang M, Ding X (2017). Role of miR-21 on vascular endothelial cells in the protective effect of renal delayed ischemic preconditioning. Mol Med Rep.

[87] Mead LE, Prater D, Yoder MC, Ingram DA. Isolation and characterization of endothelial progenitor cells from human blood. Curr Protoc Stem Cell Biol. 2008;Chapter 2:Unit 2C.1. 10.1002/9780470151808.sc02c01s6. 10.1002/9780470151808.sc02c01s6 18770637

[88] Barnes TC, Anderson ME, Moots RJ (2011). The many faces of interleukin-6: the role of IL-6 in inflammation, vasculopathy, and fibrosis in systemic sclerosis. Int J Rheumatol.

[89] Salajegheh A, Salajegheh A (2016). Urokinase plasminogen activator. Angiogenesis in health, disease and malignancy Springer.

[90] Basire A, Sabatier F, Ravet S, Lamy E, Mialhe A, Zabouo G (2006). High urokinase expression contributes to the angiogenic properties of endothelial cells derived from circulating progenitors. Thromb Haemost.

[91] Margheri F, Chillà A, Laurenzana A, Serratì S, Mazzanti B, Saccardi R (2011). Endothelial progenitor cell-dependent angiogenesis requires localization of the full-length form of uPAR in caveolae. Blood..

[92] Kiyan Y, Tkachuk S, Hilfiker-Kleiner D, Haller H, Fuhrman B, Dumler I (2014). oxLDL induces inflammatory responses in vascular smooth muscle cells via urokinase receptor association with CD36 and TLR4. J Mol Cell Cardiol.

